# Complete genome sequence of *Pseudomonas* sp. PK-RTE-24 isolated from lettuce

**DOI:** 10.1186/s13104-026-07867-7

**Published:** 2026-05-11

**Authors:** Chahrazed Belhout, Claudia Aldeia, Andrea Endimiani

**Affiliations:** Institute for Infectious Diseases, University of Bern, Friedbühlstrasse 25, CH-3001 Bern, Switzerland

**Keywords:** *Pseudomonas*, β-lactamases, MALDI-TOF MS, Whole-genome sequencing, Taxonomy, Lettuce

## Abstract

**Objectives:**

Fresh vegetables can harbor diverse bacteria, including *Pseudomonas* spp. In routine diagnostics, isolates are often identified by MALDI-TOF MS, but novel or unreferenced taxa can be misidentified. Here, we report the WGS of *Pseudomonas* sp. strain PK-RTE-24, initially assigned by MALDI-TOF MS to a known species. The aim was to generate genomic data that clarify the taxonomic assignment of PK-RTE-24 that is not linked to any described species and to highlight the discrepancy between MALDI-TOF MS identification and WGS analysis.

**Data description:**

PK-RTE-24 was isolated from ready-to-eat lettuce during screening for extended-spectrum β-lactamase (ESBL)-producing bacteria. MALDI-TOF MS reported *Pseudomonas koreensis.* Using Illumina and Oxford Nanopore technologies a complete 5,847,647-bp genome, with 60.04% GC content, and encoding 5,285 predicted genes, was assembled. Phylogenomic analysis and average nucleotide identity showed that PK-RTE-24 did not match any described *Pseudomonas* sp. and fell below the species threshold, grouping within an environment-associated clade. Antimicrobials testing revealed β-lactams non-susceptibility, consistent with a chromosomally-encoded class C β-lactamase (AmpC) gene (1,167-bp). The 388 amino-acid protein shared 96.9% identity with an AmpC from *Pseudomonas* sp. RG1. The PK-RTE-24’s genome sequence provides valuable data for classifying environment-associated *Pseudomonas* spp. and elucidating β-lactam resistance in non-clinical strains.

## Objective

The growing consumption of fresh vegetables has raised concerns regarding microbial contamination and antimicrobial resistance in environment-associated bacteria [1]. *Pseudomonas* spp. are commonly detected on leafy greens, such as lettuce, and include species with spoilage potential or opportunistic pathogenicity [2]. In this context, food and environmental microbiology laboratories rely on MALDI-TOF MS, which provides rapid species identification results but is limited by incomplete reference libraries [3, 4].

In this work, we describe *Pseudomonas* sp. strain PK-RTE-24, a resistant isolate recovered from a retail ready-to-eat lettuce sample. MALDI-TOF MS initially identified the strain as a known *Pseudomonas* species. However, whole-genome sequencing (WGS) analysis based on average nucleotide identity (ANI) and digital DNA–DNA hybridization (dDDH) revealed that PK-RTE-24 fell below the species-level thresholds when compared with described *Pseudomonas* spp., indicating that it represents a previously undescribed species.

Therefore, our objective was to sequence the genome of PK-RTE-24 to clarify its taxonomic position and provide a genomic resource for comparative and functional studies.

## Data description

Strain PK-RTE-24 was isolated from the phosphate-buffered saline (PBS) rinse of ready-to-eat lettuce purchased from a Swiss supermarket (2024). The rinse was plated on a ChromID ESBL plate (bioMérieux) following a 24-h incubation at 37 °C. PK-RTE-24 was initially identified as *P. koreensis* using the MALDI-TOF MS (MALDI Biotyper, Bruker Daltonics; FlexControl v3.4 [build 135.14]) with a score of 2.2. Antimicrobial susceptibility tests (ASTs) were performed using broth microdilution Sensititre GNX2F and ESB1F panels (Thermo Fisher Scientific) and interpreted according to the 2025 EUCAST criteria for *Pseudomonas* spp. (https://www.eucast.org/; version 15.0).

Genomic DNA (gDNA) was extracted from colonies grown overnight at 37°C on a CSBA (Columbia Sheep Blood Agar) plate (Oxoid) using the PureLink Microbiome DNA Purification Kit (Thermo Fisher Scientific) and sequenced on the Illumina NovaSeq 6000 (2 × 150-bp paired-end) and Oxford Nanopore Technologies (ONT) MinION Mk1B (SQK-RBK114.24 kit; FLO-MIN114 R10.4.1 flow cell). Illumina reads were adapter-trimmed using Trimmomatic v0.39 and quality-controlled with Qualimap2 v2.2.2. ONT reads were adapter-trimmed with Porechop v0.2.4, quality-filtered and controlled with Filtlong v0.2.1 and NanoStat v1.6.0, respectively [5–8]. Long reads were assembled using Autocycler v0.2.0. The assembly was polished with Illumina reads using Polypolish v0.6.0 [9, 10]. To verify the robustness and structure of the results, a hybrid assembly was also generated and compared using Unicycler v0.5.1 [11]. Quality assessed with QUAST v5.2.0 and CheckM2 v1.0.2, and annotated using Bakta v1.11.3 [12–14]. Genome-based taxonomy of PK-RTE-24 was determined using the TYGS platform (https://tygs.dsmz.de/, accessed on November 10^th^, 2025) by comparing its genome to related species with dDDH, later confirmed by ANI using OrthoANIu [15, 16]. A genome distance-based tree was generated from dDDH values calculated using TYGS and visualized with iTOL v7.2.2. Antimicrobial resistance genes (ARGs) were screened using AMRFinder v4.0.23 (50% minimum identity) and ResFinder v4.7.2 [17, 18]. Default parameters were used for all software unless otherwise specified. Protein sequences of described *Pseudomonas*-derived cephalosporinases (PDCs) were downloaded from NCBI GenBank, aligned using MAFFT v7.490 (L-INS-i), and visualized in Geneious Prime v2025.2.1.

Sequencing generated 6,645,947 paired-end reads and 301,337 long reads (N50, 8,338-bp). The assembly consisted of a 5,847,647-bp circular chromosome, with 333x coverage depth, 98.95% completeness, 0.02% contamination, and 60.04% GC content. Bakta annotated 5,285 genes (5,135 CDS and 150 RNA genes).

Genome-based analysis based on dDDH placed PK-RTE-24 within the *Pseudomonas* genus, clustering in an environment-associated clade (Data file 1) [19]. The closest described match was *P. siliginis* SWRI31^T^, with a dDDH value of 69%, below the established species delineation threshold (≥70%) [20]. PK-RTE-24 showed 79.7% dDDH with *Pseudomonas* sp. Leaf434 (GCF_001425545.1), an undescribed strain recovered from *Arabidopsis thaliana* leaf surfaces [21]. ANI analysis further validated this result (species demarcation >95%) [22]. Specifically, PK-RTE-24 exhibited 96.2% homology with *P. siliginis* SWRI31^T^ and only 87.1% with *P. koreensis* DSM 16610^T^, whereas similarity reached 97.5% ANI with Leaf434. For complete comparative context, Leaf434 also yielded 96.26% ANI and 69.0% dDDH when compared to *P. siliginis* SWRI31^T^. Together, dDDH and ANI values demonstrated that PK-RTE-24 was distinct from all described *Pseudomonas* spp., but belonged to the same species as the unidentified strain Leaf434. This discrepancy between MALDI-TOF MS and genome-based classification is due to the lack of respective spectra in the database of our MALDI-TOF MS.

Phenotypically, PK-RTE-24 exhibited β-lactams non-susceptibility (Data file 2) [23], compatible with the production of an intrinsic chromosomal AmpC gene (1,167-bp). The protein (388 amino-acids) shared 96.9% identity with an AmpC from *Pseudomonas* sp. RG1 (WP_264365510), an environmental isolate recovered from supermarket alfalfa sprouts. Alignment against described PDCs showed only ~60% amino-acid identity, despite the presence of conserved AmpC motifs [24]. This low similarity indicated that the enzyme did not belong to the PDC family, but represented a distinct AmpC lineage (Data file 3) [25]. This divergence further supported the genomic distinctiveness of PK-RTE-24 and its placement as a putative novel *Pseudomonas* species.

The data were deposited in Figshare and the NCBI database (Table 1) [19, 23, 25, 26].

**Table 1:** Overview of data files/data sets

Label	Name of data file/data set	File type (file extension)	Data repository and identifier (DOI or accession number)
Data file 1	Genome distance-based tree constructed from digital DNA–DNA hybridization (dDDH) values calculated using the TYGS platform. Bootstrap support values are indicated at the nodes. The tree was visualized and annotated using iTOL. *Pseudomonas* sp. PK-RTE-24 and *Pseudomonas* sp. Leaf434 are highlighted in bold.	PDF file (.pdf)	Figshare (10.6084/m9.figshare.30800045) [19]
Data file 2	Antimicrobial susceptibility profile for *Pseudomonas* sp. strain PK-RTE-24	Spreadsheet (.xlsx)	Figshare (10.6084/m9.figshare.30819161) [23]
Data file 3	Multiple sequence alignment of the class C β-lactamase from *Pseudomonas* sp. PK-RTE-24, *Pseudomonas* sp. RG1, and representative *Pseudomonas*-derived cephalosporinase (PDC) variants. The alignment was MAFFT v7.490 (L-INS-i) and visualized in Geneious Prime v2025.2.1.	PDF file (.pdf)	Figshare (10.6084/m9.figshare.30851099) [25]
Data set 1	Complete genome sequence of *Pseudomonas* sp. PK-RTE-24	Fasta file (.fna)	NCBI GenBank accession number:CM133224 (http://identifiers.org/nucleotide:CM133224.1) [26]
Data set 2	Raw sequence reads of *Pseudomonas* sp. PK-RTE-24	Fastq (.fastq.gz)	NCBI SRA Illumina reads (http://identifiers.org/ncbiprotein:SRR37455609)/ Nanopore reads (http://identifiers.org/ncbiprotein:SRR37455608) [27]

## Limitations

Although the genome sequence of *Pseudomonas* strain PK-RTE-24 is complete and of high quality, no species designation is currently assigned. Strict application of the <70% dDDH threshold indicates that PK-RTE-24 and its closest relative, Leaf434, represent a putative novel species or subspecies distinct from, but closely related to, *P. siliginis*. It should be noted that the available reference genome for *P. siliginis* SWRI31^T^ is a draft assembly, which provides important context for the borderline dDDH values (69.0%). Formal species designation will require additional phenotypic, biochemical, and chemotaxonomic characterization.

The discrepancy between MALDI-TOF MS and genome-based identification likely reflects the limited representation of environmental *Pseudomonas* taxa in current spectral libraries, which restricts confident species-level resolution in routine settings.

## Data Availability

The genome of PK-RTE-24 has been deposited in GenBank under accession number [CM133224] (http:/identifiers.org/nucleotide:CM133224.1) [26]. The raw sequencing reads have been deposited in the NCBI Sequence Read Archive (SRA) under accessions [SRR37455609] (https:/www.ncbi.nlm.nih.gov/sra/?term=SRR37455609) (http://identifiers.org/ncbiprotein:SRR37455609) (Illumina) and [SRR37455608] (https:/www.ncbi.nlm.nih.gov/sra/?term=SRR37455608) [http://identifiers.org/ncbiprotein:SRR37455608] (Oxford Nanopore). The BioProject and BioSample accession numbers are [PRJNA1359604] (https:/www.ncbi.nlm.nih.gov/bioproject/PRJNA1359604) [http://identifiers.org/bioproject:PRJNA1359604] and [SAMN53172773] (https:/www.ncbi.nlm.nih.gov/biosample/?term=SAMN53172773) (http://identifiers.org/biosample:SAMN53172773), respectively. Additional data files supporting the findings of this work, including the genome distance-based tree [10.6084/m9.figshare.30800045] [19], the antimicrobial susceptibility profile [10.6084/m9.figshare.30819161] [23], and the class C β-lactamase multiple sequence alignment [10.6084/m9.figshare.30851099] [25]. All processed files are hosted on Figshare and freely accessible under a CC-BY 4.0 license. See Table 1 for details and links to the data.

## Abbreviations

AmpC: Class C β-lactamase
ANI: Average nucleotide identity
ARGs: Antimicrobial resistance genes
ASTs: Antimicrobial susceptibility tests
bp: Base pair
CSBA: Columbia sheep blood agar
dDDH: Digital DNA–DNA hybridization
ESBL: Extended-spectrum β-lactamase
gDNA: Genomic DNA
MALDI-TOF MS: Matrix-assisted laser desorption/ionization time-of-flight mass spectrometry
MIC: Minimum inhibitory concentration
ONT: Oxford nanopore technology
PBS: Phosphate-buffered saline
WGS: Whole-genome sequencing
